# Making Millennial Medicine More Meta

**DOI:** 10.1128/mSystems.00154-17

**Published:** 2018-03-06

**Authors:** Peter J. Turnbaugh

**Affiliations:** a Department of Microbiology & Immunology, UCSF, San Francisco, California, USA

**Keywords:** chemical biology, metagenomics, microbiome, pharmacology, precision medicine, toxicology

## Abstract

Although the importance of human genetic polymorphisms in therapeutic outcomes is well established, the role of specific genotypic or copy number variants in our “second genome” (the microbiome) has been largely overlooked. In this Perspective, I will discuss three major barriers to integrating metagenomics into pharmacology, highlighting ongoing research by us and others that has begun to shed light on the mechanisms that link the human microbiome to the efficacy and toxicity of small-molecule and biological therapies. The challenges for the next 5 years and beyond are many, requiring interdisciplinary scientific teams working at the interface of chemistry and biology, and a consideration of far more variables than traditionally included in pharmacological modeling. However, the potential benefits are immense. Continued progress could enable more precise tools for predicting patient responses and the development of a new generation of therapeutics based on, or targeted at, the human microbiome.

## Abstract (precis)

Although the importance of human genetic polymorphisms in therapeutic outcomes is well established, the role of specific genotypic or copy number variants in our “second genome” (the microbiome) has been largely overlooked. In this Perspective, I will discuss three major barriers to integrating metagenomics into pharmacology, highlighting ongoing research by us and others that has begun to shed light on the mechanisms that link the human microbiome to the efficacy and toxicity of small-molecule and biological therapies.

## PERSPECTIVE

Over the past decade, the emerging microbiome field has emphasized the tremendous phylogenetic and metabolic diversity found within the complex microbial communities that live in and on humans and other mammals. These microbiomes profoundly extend host metabolism with widespread consequences for the metabolism of endogenous and dietary compounds (1). But one of the most surprising findings to date is that the human microbiome also metabolizes xenobiotics, compounds foreign to the body, including drugs (both synthetic and natural products), food additives, and environmental toxins (2, 3). This fact is counterintuitive when one considers the competitive and dynamic nature of host-associated microbial communities. Why and how would host-associated microorganisms evolve mechanisms to metabolize compounds that they do not normally encounter? Even the most successful drugs are only used for short periods of time or in a selected subset of the population. While microbes may gain a transient fitness advantage from xenobiotic metabolism, this selection pressure would likely be insufficient to maintain these genes over longer timescales. Alternatively, perhaps these biotransformations are indicative of a broader substrate scope of microbiome-encoded enzymes that evolved to metabolize compounds typically found within the body.

Our ongoing research program seeks to address these fundamental scientific questions while also establishing the feasibility of translating microbiome research to improve the treatment of human disease. Unpredictable variations in drug response are a major limitation of modern medicine. While one patient may show a miraculous recovery, the next may show very little response or have an adverse drug outcome. Polymorphisms in the human genome are certainly important, but they often fail to explain most of the observed variation in treatment outcomes—currently <1% of drugs are dosed on the basis of genetic information. A primary reason for poor drug response is incomplete absorption or bioavailability. That is, the drug is metabolized prior to entering systemic circulation or intestinal efflux transporters reduce its absorption. Our overarching hypothesis is that the human microbiome represents an underappreciated contributor to interindividual variations in drug bioavailability. Here, I will discuss three major challenges in this area and the ongoing efforts by us and others to address them.

**FIG 1:**
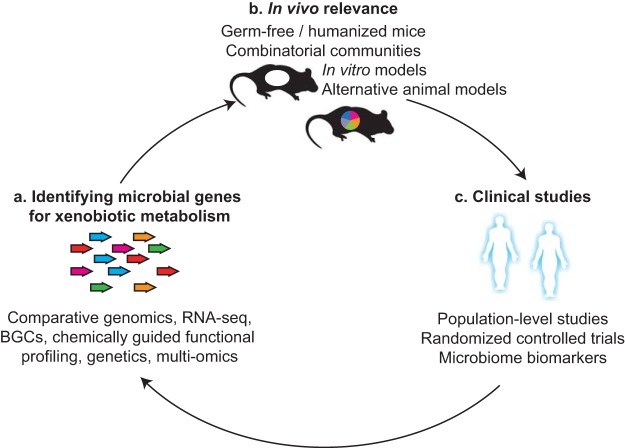
Three major barriers to the integration of metagenomics into pharmacology and toxicology. (a) The microbial genes responsible for the metabolism of xenobiotics (foreign compounds, including synthetic and natural-product drugs, food additives, and environmental toxins) remain poorly understood. Established methods for linking novel genes to functions include comparative genomics, transcriptional profiling (RNA-seq), the computational prediction of biosynthetic gene clusters (BGCs), and chemically guided functional profiling. Genetic tools are still under development for most members of the human microbiome, including traditional mutagenesis and more advanced genome editing. Advances in multiomics will enable systematic links among genes, transcripts, proteins, and metabolites. (b) Proving the *in vivo* relevance of specific microbial strains, genes, or products will require continued refinement and application of methods for the study of gnotobiotic mice, *in vitro* systems, and alternative animal models (e.g., fish, worms, and pigs). (c) The gold-standard clinical trial design (randomized controlled trials) have only recently begun to be applied to the human microbiome, especially in the context of pharmacology. These studies will help to make stronger claims about causation in large-scale population-based studies and enable the identification of mechanistic microbiome-based biomarkers of drug response. Better integration of data from all three approaches is critical.

The first major barrier to using sequencing-based metagenomics to study microbial drug metabolism is that the genes responsible remain unknown or poorly characterized (Fig. 1a). Recent studies have begun to address this knowledge gap even for human-associated bacteria that lack genetic tools (i.e., most of them). We have demonstrated that transcriptional profiling (transcriptome sequencing [RNA-seq]) is a valuable and rapid tool for identifying genes involved in drug metabolism from gut bacterial isolates (4, 5) and even complex distal gut microbial communities (6). Comparative genomics represents a complementary approach because of the observation that closely related bacterial strains often vary in their metabolism of drugs and other substrates of interest. For example, we identified a genomic locus missing from strains of *Eggerthella lenta* incapable of metabolizing the cardiac drug digoxin and significantly upregulated in response to the drug (4). It is also possible to directly mine metagenomic data sets for novel metabolic activities by searching for biosynthetic gene clusters (7) or through “chemically guided functional profiling” (8). The latter approach begins with the quantification of the abundance and prevalence of protein families and subfamilies, which can then be used to generate testable hypotheses about enzyme function. Continued progress will require leveraging more advanced tools for bacterial genetics, including clustered regularly interspaced short palindromic repeat(s)-Cas-based genome editing, enabling the application of a chemical genomic approach to complex microbial communities. Improvements in the experimental and computational methods for integrating “multiomics” data sets (metagenomics, -transcriptomics, -proteomics, and -metabolomics) could also enable more systematic links between genes coding for proteins of unknown function and specific biotransformations.

The second major barrier to progress in this area is that most of the research on host-associated microbial drug metabolism has focused on *in vitro* systems that are far removed from the microenvironment and microbial diversity where these metabolic pathways operate or *in vivo* models where precise manipulation of the microbiome is currently impossible. For example, antibiotic depletion was used to link the gut microbiome to the bioavailability and response to the nonsteroidal anti-inflammatory drug indomethacin (9) and cohousing experiments implicated the gut microbiome in antitumor immunity (10). These seminal studies have now set the stage for follow-on experiments using *in vitro* approaches for building complex microbial communities (so-called synthetic ecologies) and testing their metabolic activity in batch culture, bioreactors, organoids, and/or organ-on-a-chip models. Parallel experiments with gnotobiotic (germfree and colonized) animals are necessary to establish *in vivo* relevance, providing a tractable model to elucidate the host and microbiome mechanisms responsible, their interdependencies, and the impact of environmental factors like dietary intake, as we have recently done for the cardiac drug digoxin (4). Together, these complementary approaches will help determine the impact of specific microbes, microbial enzymes, and microbial products on drug pharmacokinetics and -dynamics, while also considering the context dependence of these factors.

Concerted efforts need to be made to accelerate and improve gnotobiotic mouse research and to expand these techniques to other preclinical animal models (Fig. 1b). A major advance was made by validating the feasibility of performing combinatorial colonization experiments wherein germfree mice are systematically colonized with combinations of microorganisms in traditional microisolator cages, decreasing the bottleneck due to the use of a single large vinyl isolator for each colonization group (11). “Humanized mice” provide another useful model for comparing the effects of different donor communities; our experiments demonstrated that germfree mice can be stably colonized with the human gut microbiome, enabling controlled experiments that would be difficult, if not impossible, to perform with human cohorts (12). However, far more work is needed to establish robust protocols that optimize engraftment efficiency (i.e., the percentage of donor strains that colonize the germfree recipient) and minimize shifts in microbial community structure and function following transplantation. Studies with mice and other rodent models will also benefit from the use of high-throughput genetic screening to identify host and microbial factors that shape drug metabolism using *Caenorhabditis elegans* (13), zebrafish, flies, and other model organisms amenable to this approach. Paired studies with multiple laboratory and even wild animals would provide valuable insights into the reproducibility and evolutionary conservation of pathways for microbial xenobiotic metabolism and host-microbiome interactions relevant to pharmacology. Differences between animals could help to generate hypotheses about the effect of host diet, anatomy, and adaptive immunity, among other factors relevant to the microbiome. These types of comparative studies, rare in the microbiome field, may also provide a compelling argument for relevance to human patients.

The third, and final, barrier to progress is that we lack data from randomized controlled trials in patient populations (Fig. 1c). One recent example of the utility of this approach comes from studies of the impact of metformin on the gut microbiomes of treatment-naive type 2 diabetes patients (14). Subjects were randomized to placebo or metformin treatment, providing more definitive evidence that metformin causally impacts the human gut microbiome. Transplantation of the posttreatment gut microbiome into germfree mice suggested that these microbial shifts may actually contribute to improvements in glucose tolerance. On a population scale, cross-sectional analyses have demonstrated that medication explains the largest total variance in gut microbial community structure (15). It remains an open question how many of these drugs directly impact the gut microbiome or if the resulting microbial signatures are reflective of drug response and/or adverse effects. Importantly, these studies imply that the full scope of drugs that are impacted by the human microbiome could extend far beyond those that are directly metabolized in the gastrointestinal tract. Drug-induced changes to the microbiome in the gut or other body habitats may represent a generalizable “off-target” effect with unanticipated consequences for treatment outcomes.

In conclusion, it is now clear that understanding the role of the human microbiome in pharmacology and toxicology is essential for clinical, economic, and scientific reasons. Variations in drug toxicity can have devastating and even fatal consequences for patients. Even in the absence of acute toxicity, failure to rapidly establish effective treatment can lead to lifelong increases in disease severity. Continued progress in this research area could enable clinicians to tailor the drug and dosage to a given patient by using microbiome-based biomarkers. Alternatively, the microbiome could be targeted by using antibiotics, small-molecule inhibitors, genome editing, or dietary interventions. The cost of drug development is escalating at an alarming rate, currently $5 billion per drug, motivating the development of more relevant preclinical models, the rational design of drugs to avoid undesirable host and microbial biotransformations, and methods to exploit the microbiome to better control the rate and location of drug delivery. Finally, this research will lead to testable hypotheses as to how and why microbial xenobiotic metabolism evolved, providing new insights into the factors that control the structure and function of the human microbiome.
